# Rare Root Morphology of a Maxillary Central Incisor Associated With Gingival Hyperplasia: An Endodontic Case Report

**DOI:** 10.1097/MD.0000000000003617

**Published:** 2016-05-06

**Authors:** Monica Monea, Cosmin Moldovan

**Affiliations:** From the Department of Odontology and Oral Pathology (MM), Faculty of Dental Medicine; and Department of Histology (CM), Faculty of Medicine, University of Medicine and Pharmacy, Tirgu Mures, Romania.

## Abstract

Dilaceration is a developmental disturbance characterized by the angulation of the crown or root of a permanent tooth, which is often related to trauma of primary dentition. We report a case of a dilacerated root in a maxillary central incisor associated with gingival hyperplasia in a patient under fixed orthodontic treatment, a combination of pathological conditions that had never been mentioned before in the scientific literature.

A 10-year-old female patient presented to the Department of Odontology and Oral Pathology with tenderness to palpation and bleeding from the oral aspect of the central incisor, alerted by the proliferation of the gingiva. During clinical examination, the palpation performed with a dental probe revealed a carious lesion with dental pulp exposure on the distal aspect of right central incisor and the presence of a sessile mass of inflamed gingival tissue that proliferated inside the defect. On the preoperative radiograph a dilacerated root canal was noted, without periapical bone resorption.

The main diagnosis was irreversible pulpitis and gingival hyperplasia and the treatment option was surgical removal of the inflamed tissue with histopathological examination and root canal treatment. Successful endodontic treatment with a good prognosis was recorded.

The measurement of the root curvature proved to be extremely helpful in choosing the right endodontic technique and made the treatment easier than expected. An important observation was that, despite the rare clinical and radiographic aspect of this dilacerated tooth, the endodontic treatment proved to be relatively easy to perform and, therefore, the prognosis was considered favorable.

## INTRODUCTION

The morphologic change in the endodontic anatomy known as dilaceration was defined in many ways in the scientific literature, based on different criteria set by the authors.^1–4^ It was considered an abrupt deviation of the long axis of the crown or root portion of a tooth due to a traumatic non-axial displacement of already formed hard tissue in relation to the developing soft tissue. This condition occurs frequently after intrusion, avulsion, and subluxations of primary teeth.^4–6^ The difficulty of dilacerated or S-shaped root canals depends on the degree of curvature and on the ability of the specialist to choose the appropriate endodontic method for cleaning and shaping, in order to prevent complications and enhance the quality of the treatment.

The purpose of this paper is to describe the endodontic management of a maxillary central incisor with an S-shaped root associated with gingival hyperplasia, with emphasis on technical procedures that allow a complete surgical removal of the enlarged soft tissue and adequate root filling, in order to prevent or reduce possible complications.

### Patient Information

A 10-year-old female patient presented to the Department of Odontology and Oral Pathology for moderate pain and bleeding during mastication in the maxillary central region. She had been under orthodontic treatment with a fixed appliance for 6 months, with metal brackets attached to the maxillary incisors. The medical history was not relevant.

### Clinical Findings

**FIGURE 1:**
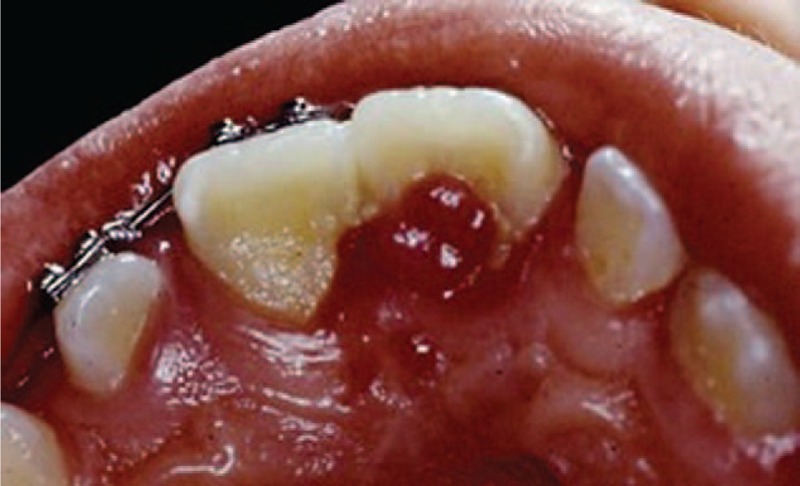
Preoperative clinical image showing heavy accumulation of oral biofilm and the inflammatory changes of the gingival tissue (gingival polyp) from the oral aspect of the tooth.

Clinical examination included inspection and probing, the latter revealing a carious lesion with dental pulp exposure on the distal aspect of right central incisor and the presence of a sessile mass of inflamed gingival tissue adjacent to the carious lesion. It was an irregular bleeding smooth surface with dark red ulcerated areas, tender to palpation, which was diagnosed as gingival hyperplasia (Figure 1).

### Diagnostic Assessment

**FIGURE 2:**
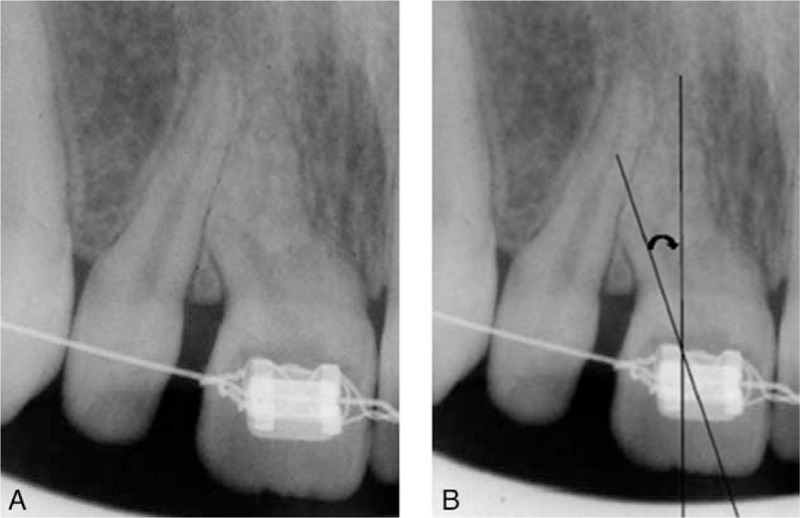
A. Maxillary central incisors; preoperative radiograph showing an S-shaped canal. B. Based on Schneider's method an angle of deviation of 20° was measured.

The differential diagnosis with a dental pulp polyp was made based on the connection to the gingiva, not to the pulp cavity, which was exposed by caries. Heavy accumulation of oral biofilm was observed on the oral aspects of the teeth. The tooth was not tender to percussion but gave a strong positive response to palpation and sensitivity tests performed with ice sticks. The radiographic examination carried out by long-cone paralleling technique on double Kodak Ektaspeed films (Kodak, Rochester, NY) revealed a double curvature of the root resembling an S-shape (Figure 2A). The curvature of the root was measured using a method described by Schneider (Figure 2B) and the angulation was estimated at 20°, measured on a computer screen, which was an indication for using hand nickel-titanium instruments and the “balanced force” technique introduced by Roane et al in 1986.^7^ Based on the clinical and radiographic examination the final diagnosis was moderate dilacerations of a maxillary central incisor with irreversible pulpitis and gingival polyp. Surgical removal with histopathologic examination of the gingival tissue was indicated, followed by endodontic root canal treatment. The prognosis was considered favorable. Prior to any medical procedure, a written consent from the parents was required and then the patient was referred for root canal treatment. The patient was scheduled for endodontic therapy.

### Therapeutic Intervention

**FIGURE 3:**
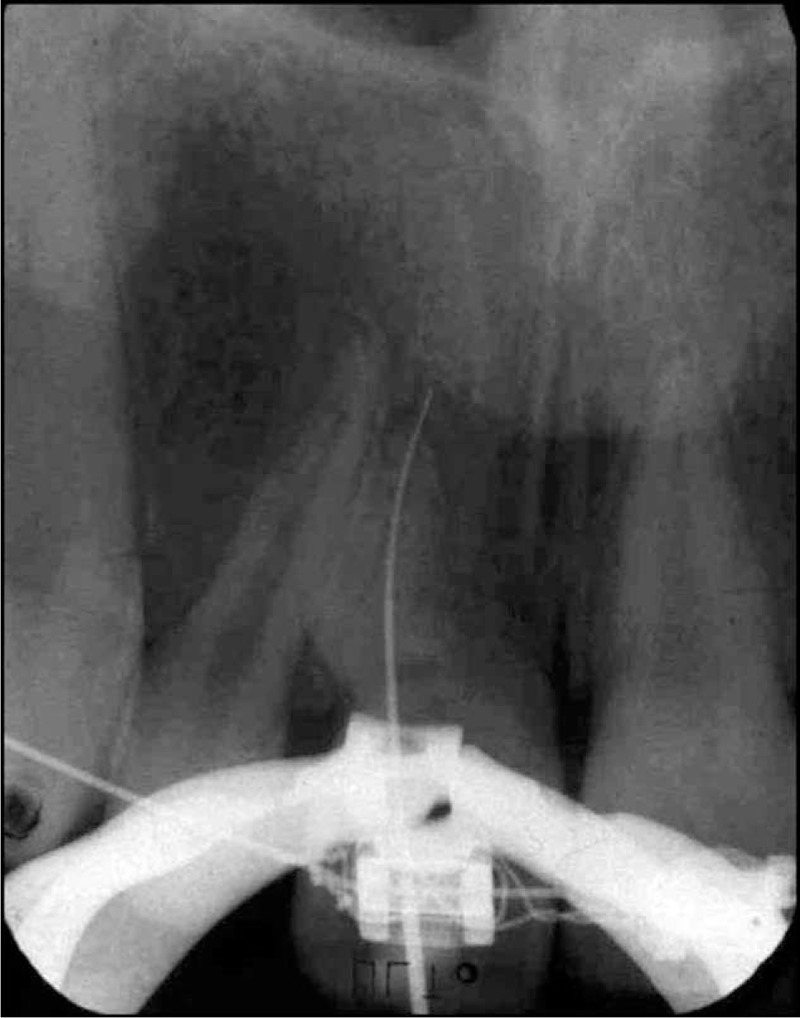
Radiographic measurement of the working length with a Kerr file #15.

**FIGURE 4:**
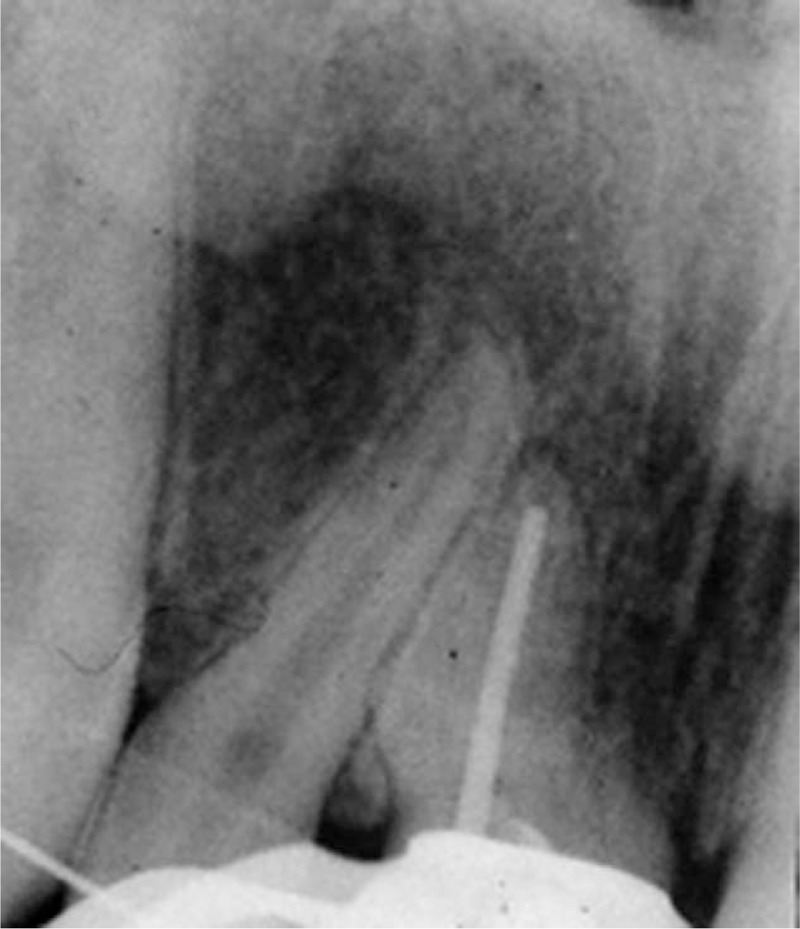
Radiographic control of the apical position of the master cone. It confirmed that the preparation was correct, reaching 1 mm from the root apex.

**FIGURE 5:**
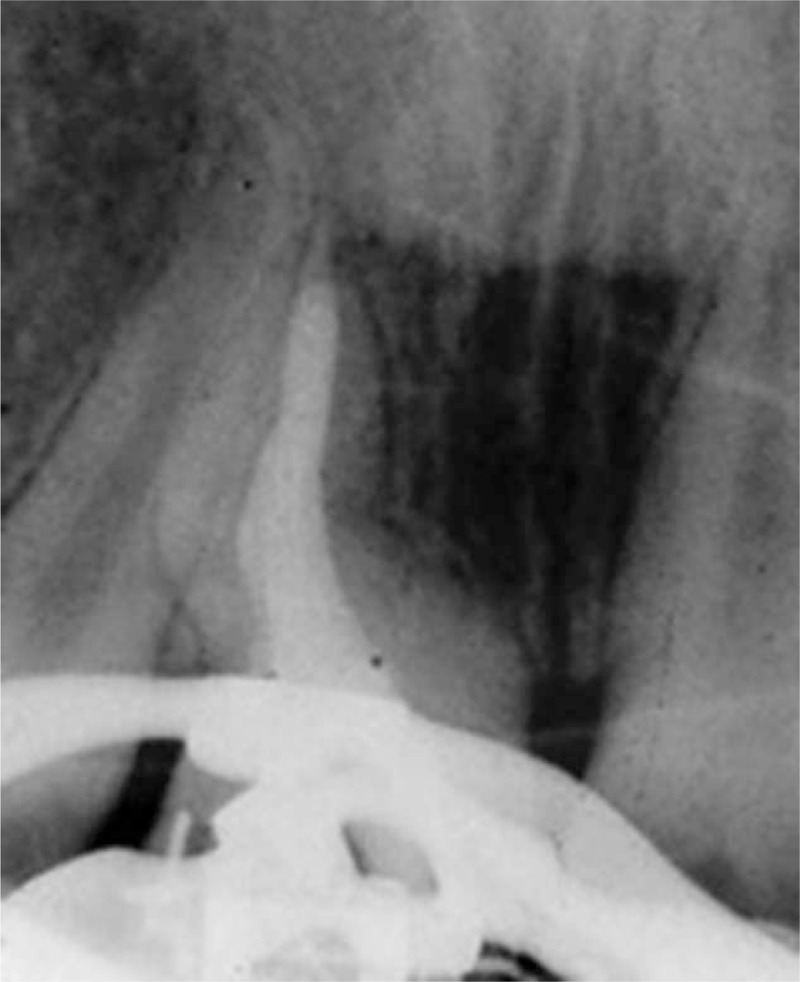
Final radiograph, control of the lateral condensation. It showed that the entire root canal space was filled with sealer and core material.

**FIGURE 6:**
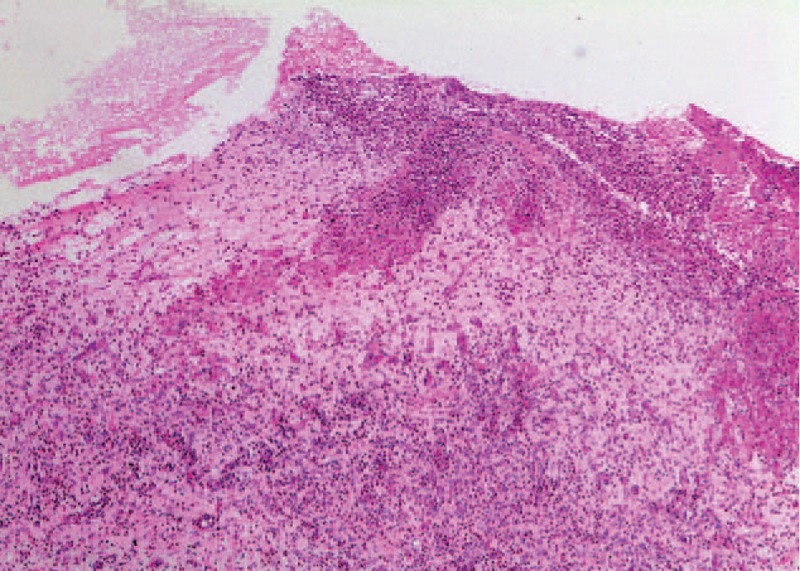
Gingival connective tissue with numerous fibroblasts and inflammatory cells. Ulcerated surface epithelium. (Hematoxylin eosin, ×4).

**FIGURE 7:**
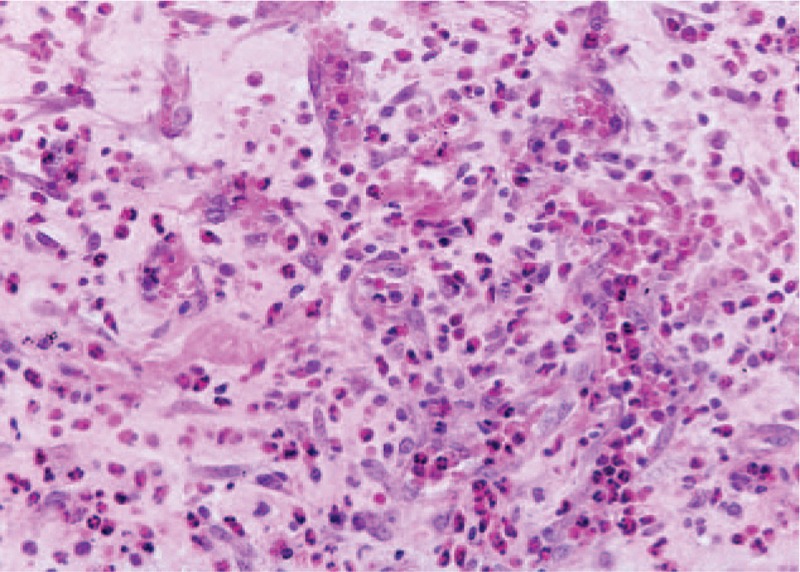
Granulation tissue, fibroblasts, and inflammatory infiltrate. (Hematoxylin eosin, ×20).

**FIGURE 8:**
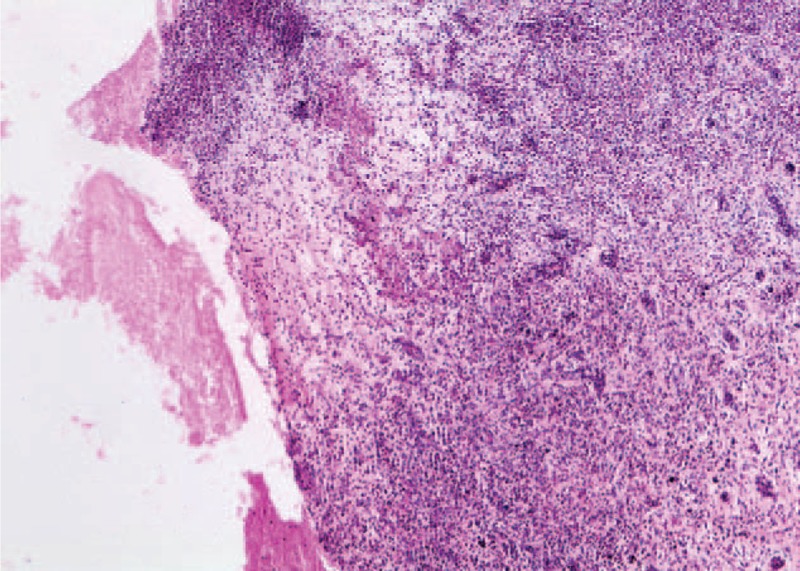
Gingival connective tissue with edema and polymorph inflammatory infiltrate. Ulceration of the surface epithelial tissue. (Hematoxylin eosin, ×4).

**FIGURE 9:**
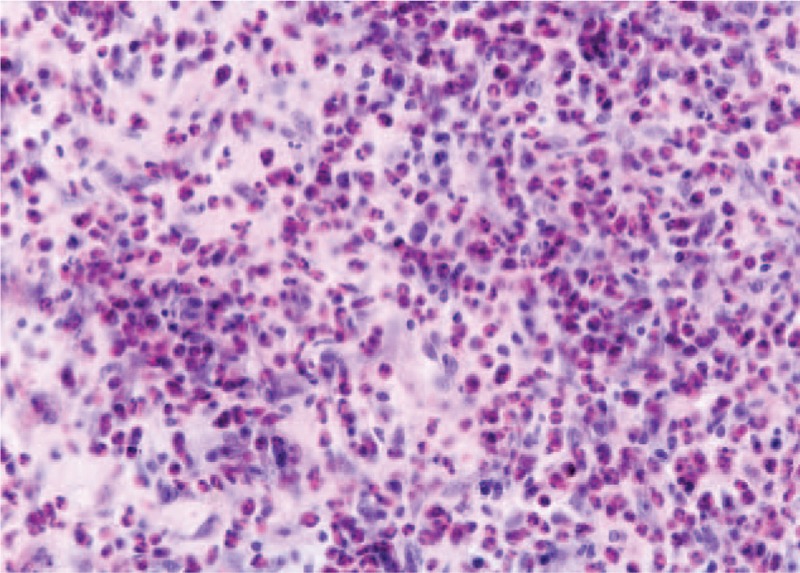
Polymorph inflammatory infiltrate, edematous areas in the gingival connective tissue. (Hematoxylin eosin, ×20).

Local anesthesia was obtained by using a topical application of 20% Lidocaine gel placed on a cotton pellet in order to reduce the local discomfort and then with 2 mL Mepivacaine 2% + 1:100,000 epinephrine (Septodont, Maidstone, Kent, UK). The gingival polyp was removed with an electrocautery instrument and sent for histopathological examination. After complete exposure of the carious lesion the endodontic treatment was carried out. Under complete isolation with rubber-dam, the coronal dental pulp was removed and the cavity was irrigated with 5 mL of 5.25% sodium hypochlorite (NaOCl) in order to expose the entrance into the root canal, followed by the removal of the entire radicular pulp. The working length was measured with a #15 instrument up to 0.5 mm from the apex, confirmed by a periapical radiograph (Figure 3). The treatment was completed with ProTaper nickel-titanium (Ni-Ti) hand files (Dentsply Maillefer, Ballaigues, Switzerland) according to the “balanced force” technique. A gutta-percha master cone #60 was placed and the fitting up to the working length was checked radiographically (Figure 4). For the root canal filling an AH Plus sealer (Dentsply, DeTrey GmbH, Konstanz, Germany) and gutta-percha points were used, according to the lateral condensation technique. The final radiographic examination (Figure 5) showed that the root filling was correct and the access cavity was closed by a temporary restoration with ionomer cement (Fuji IX, GC Corp) and the patient received postoperative instructions. The histological evaluation of the gingival tissue is presented in Figures 6–9. The histological examination of our specimen showed the presence of a granulation tissue with a rich inflammatory infiltrate, numerous fibroblasts in the connective tissue and ulcerated zones in the epithelial surface layer.

### Follow-Up and Outcome

The patient followed the oral hygiene instructions in order to promote the gingival healing. The tooth was asymptomatic and after 14 days a permanent composite restoration was placed.

## DISCUSSION

The evaluation of the root curvatures is of utmost importance in order to choose the appropriate endodontic technique. In this way, complications as root perforations are diminished and the probability of a favorable clinical result is increased. The endodontic treatment in this case was carried out after we had measured the root curvature; based on the result (moderate) we have chosen the most appropriate instruments and technique. Finally, this proved to be easy, economic and suitable for high quality results and excellent long-term success in endodontic treatment. A limitation in this case was the lack of a cone beam computed tomography investigation, due to financial reasons, which could give us a 3-dimensional image of the root anatomy. Knowledge of dental anatomy and its variations is essential for the success of endodontic treatment. A clinician is required to have an insight of the morphology of tooth related to its shape, form, and structure before commencing the treatment. The soft tissues surrounding the dental structures are also important, as they can interfere with the diagnosis or the endodontic therapy. On occasion, the gingival tissue adjacent to a broken carious tooth may proliferate into the defect and a gingival polyp is formed, frequently resembling a hyperplastic pulpitis. The distinction is made based on careful examination of the tissue mass, to determine if it is connected to the pulp or gingiva. In a study on 530 cases of focal reactive lesions of the oral cavity Hunagsi et al^8^ found that inflammatory gingival hyperplasia was the most prevalent lesion with 51% of the cases. The extent of the root curvature is one of the most important variables that could lead to instrument fracture during endodontic treatment. In clinical conditions two curves can be present in the same root canal; this type of morphology is called S shape and represents a challenging condition for dental specialists.^3,7^ A major advance in imaging techniques is the introduction of cone beam computed tomography in endodontic treatment, which offers a three dimensional view of the root morphology. In this case, it was not considered necessary, as the root curvature was evident in conventional radiographic examination. The first step during endodontic treatment is the preoperative radiographic examination, which gives important information about the morphology of the roots and root canals. In this case, the root curvature was evaluated according to the method proposed by Schneider based on an angle that is obtained by two straight lines.^9^ The first is parallel to the axis of the root canal curvature and the second passes through the apical foramen until intersecting with the first line. The angle is named according to the degree of the curvature as straight (5°), moderate (10°–20°), or severe (25°–70°). In this case, the angle was measured on a computer screen at a value of 20°, which is considered moderate.

The tapered preparation of the curved canals is the ultimate challenge in Endodontics and a meticulous technique will provide sufficient enlargement of the curved canals.^10^ The final result will be influenced by endodontic instruments and technique and hardness of dentin.^3^ A significant advancement in root canal preparation with hand instruments were made by the introduction of the balanced force technique by Roane in 1986,^7^ which proved to be useful in the management of curved canals. Benefits of balanced force technique are represented by a reduced amount of debris forced in the periapical space, maintenance of the instruments in the center of the root canal, and less iatrogenic danger. Common cases of failure are related to procedural errors such as ledges, fractured instruments, canal blockages, zip, and elbow creations. The S-shaped canal has two curves, with the apical curve being very difficult to prepare; the chances of strip perforations are very high in these root canals.^6^ In this case, the balanced force technique and hand endodontic Ni-Ti files were used, as they proved to be the best choices in dilacerated root canals. The curvature was moderate and the angle was reduced by flaring the access cavity, which made the approach of the rest of the canal easier. The association of the gingival polyp was probably related to the presence of long-standing local irritation of the soft tissue, adjacent to a carious lesion and aggravated by the presence of fixed orthodontic appliance. Severe root curvature may pose substantial difficulty in cleaning and shaping as well as filling of the root canal. The lateral condensation technique used in this case was extremely efficient in filling the whole endodontic space, without any gaps between the root filling and dentin walls. The use of better imaging techniques as the cone beam computed tomography could improve the clinical diagnosis and technical performance in difficult cases.

### Patient Perspective

The long-term prognosis for this case was considered to be very good. The patient was informed about the complications that could be related to inadequate oral hygiene maintenance program during the orthodontic treatment with fixed appliance or to coronal restoration fracture, which could expose the root filling to microbial marginal leakage from the oral cavity.

### Informed Consent

An informed consent from the legal representatives of the patient was requested prior to any treatment procedure.
